# The Book World of Medicine and Science

**Published:** 1903-12-19

**Authors:** 


### The Book World of Medicine and Science.

The After-Treatment of Operations. By P. Lockhart Mummery, M.B., F.R.C.S. (London: Baillière, Tindall and Cox. 1903. Pp. 221. Illustrations 29. Price 5s. net.)

This work is to be welcomed. There is but little in the ordinary text-books on the after-treatment of operations, and often house-surgeons and practitioners are at a loss as to the best method of dealing with cases when left in their hands after the operating surgeon has finished his work. After a short introductory chapter on posture, sleeplessness, pain, etc., subsequent to an operation, the author proceeds to discuss the after-treatment of the actual wound. Such complications as hæmorrhage, shock, collapse, and thrombosis, are all adequately reviewed, as well as post-anæsthetic disturbances, and the various post-operative rashes and drug poisonings. The succeeding chapters deal with the methods in which cases should be treated after operations in the several regions of the body in particular. It is to be regretted that the writer still defines recurrent hæmorrhage as that "which occurs within twenty-four hours of the operation." Such arbitrary definitions are apt to be very misleading. It is much more scientific to speak of recurrent hæmorrhage as that which is due to the failure of nature's or the surgeon's methods of *temporary* arrest. Again the definition of secondary bleeding is equally poor, and several of the causes mentioned are in reality those which induce recurrent hæmorrhage rather than secondary. The temperature of the water to be used in flushing bleeding cavities is not clearly indicated, such terms as "warm," "hot," "really hot," being employed. The description of the treatment of shock, and its effects could hardly be improved upon. There is a good differential diagnosis between a septic and a scarlatinal post-operative rash. The after-treatment of laparotomy is fully dealt with, and some very sound and useful hints given. Beef-tea, as usually made, is denounced as it properly should be; excellent as a stimulant, practically useless as a food. Also it is good to see the semi-cruelty of "sips of water" condemned. It is very questionable whether the statement that "suppuration around a deep or buried stitch is not always the result of the suture material being septic" is in fact a true one. In dealing with hernia, the expression "a real radical cure" is an unfortunate one. The advice that a patient should be measured for an artificial limb as early as a month after amputation has been performed is not sound, and much disappointment would result if it were universally followed. The work ends with an appendix on rectal, nasal, and subcutaneous feeding, on diets, artificial limbs, etc. There is much to recommend in the book, and it is sure to obtain a place in practical surgical literature.

Handbook of Surgical Anatomy. By G. A. Wright, M.B., F.R.C.S., and C. H. Preston, M.D., F.R.C.S., L.D.S. (London: Sherratt and Hughes. 1903. Price 4s. 6d. net. Pp. 202.)

A knowledge of anatomy from a clinical point of view is of immense service in the practice of both physician and surgeon. This work is written from the surgical standpoint and contains much which is likely to be of interest and assistance. It commences with the upper extremity at the end of the fingers, works up through this limb to the neck, face and head, and so to the thorax, abdomen, perineum, pelvis, and finally down the lower extremity. In the margin of the pages the matter under consideration is indicated in heavy type, rendering reference remarkably facile. There is no attempt at any lengthy description of the structures, but the essential points are graphically stated, and the reader obtains a clear idea of the subject matter. The details given are very accurate, a somewhat closer inspection than usual failing to discover but a few errors. On page 51 it is probably meant that the left innominate *vein* is in danger from a too low incision for tracheotomy, for the innominate *artery* can hardly be said to cross the trachea. There is a little confusion in some of the terms used in connection with hernia. If an inguinal hernia be defined as a "peritoneal sac and its contents in the inguinal region" it is not correct to say that the peritoneum forms a "covering" of such a hernia. "Infantile" and "encysted" herniæ are not one and the same. Again, it is not so common for a femoral hernia to pass upwards and outwards after it has transgressed the saphenous opening. Further it is not accurate to describe at one place Scarpa's triangle as reaching at its apex as low as the junction of the upper with the middle third of the thigh, and on the next page to state that the apex is only a hand's breadth below Poupart's ligament. A comparison of these two points will prove that they do not correspond. There are no illustrations in the book. Diagrams always, if correct, help descriptions of anatomy; but unless they are numerous and well drawn it is perhaps best to delete them entirely. The book ends with a good index. We can heartily recommend the volume to students, and especially to those preparing for a final examination in surgery.

Elements of Surgical Diagnosis. By A. Pearce Gould, M.S., F.R.C.S. (London: Cassell and Co., Limited. 1903. Third Edition. Pp. 607. Price 7s. 6d.)

Mr. Pearce Gould's well-known manual for the surgical wards has reached a third edition, which is as excellent and even surpasses its predecessors. The whole text has been revised, and sections on the diagnosis of intracranial complications of otitis media, of abdominal tumours, and of certain acute abdominal affections, have been added, and will prove of great service. Perhaps nothing so clearly indicates the degree of clinical experience of a practitioner as the method in which an examination of a case is carried out, and the rapidity and accuracy of the diagnosis. A study of the pages of this book will enable the student to gather such knowledge of careful method as to provide him with the means of a quick and certain diagnosis, and save him from many an error. It is to be expected that this edition will have a wide circulation and obtain much appreciation.

